# Resolving the orthographic ambiguity during visual word recognition in Arabic: an event-related potential investigation

**DOI:** 10.3389/fnhum.2013.00821

**Published:** 2013-12-02

**Authors:** Haitham Taha, Asaid Khateb

**Affiliations:** ^1^The Unit for the study of Arabic language, Edmond J. Safra Brain Research Center for the Study of Learning Disabilities, Faculty of Education, University of HaifaHaifa, Israel; ^2^Department of Learning Disabilities, Faculty of Education, University of HaifaHaifa, Israel; ^3^The Cognitive Laboratory for Learning and Reading Research, Sakhnin College for Teachers' EducationSakhnin, Israel

**Keywords:** Arabic orthography, pseudohomophones, orthographic decision, N170 component, P2 component, P600 component, source localization

## Abstract

The Arabic alphabetical orthographic system has various unique features that include the existence of emphatic phonemic letters. These represent several pairs of letters that share a phonological similarity and use the same parts of the articulation system. The phonological and articulatory similarities between these letters lead to spelling errors where the subject tends to produce a pseudohomophone (PHw) instead of the correct word. Here, we investigated whether or not the unique orthographic features of the written Arabic words modulate early orthographic processes. For this purpose, we analyzed event-related potentials (ERPs) collected from adult skilled readers during an orthographic decision task on real words and their corresponding PHw. The subjects' reaction times (RTs) were faster in words than in PHw. ERPs analysis revealed significant response differences between words and the PHw starting during the N170 and extending to the P2 component, with no difference during processing steps devoted to phonological and lexico-semantic processing. Amplitude and latency differences were found also during the P6 component which peaked earlier for words and where source localization indicated the involvement of the classical left language areas. Our findings replicate some of the previous findings on PHw processing and extend them to involve early orthographical processes.

## Introduction

Visual word recognition in alphabetic orthographies is thought as a multi-sequential process in which different sub-processes occur within determined time windows (McClelland and Rumelhart, 1981; Rumelhart and McClelland, 1982; Bentin et al., 1999; Dehaene et al., 2005; Martin et al., 2006). These time windows represent different stages of information processing, which in written words correspond to the orthographic, phonological, and lexico-semantic ones (Salmelin et al., 1996; Pammer et al., 2004). Different theoretical models tried to describe the sequence of occurrence of such cognitive processes during visual word recognition. In the dual route models, where word familiarity and frequency play a major role (Coltheart, 2005), reading non-familiar words is supposed to rely on phonological decoding strategies (the non-lexical route), while reading familiar ones passes through the orthographic knowledge only (lexical route). Accordingly the semantic access may occur either after the phonological decoding takes place or directly using the orthographic-lexical access. Contrary to the dual route model, other models postulate the existence of one single route which allows a direct access from orthography to phonology and then into semantics (Plaut et al., 1996; Seidenberg et al., 1996). Yet, the different models agree with the notion that the process of visual word recognition begins with processing the orthographic features of the written words. In this regard, it is important mention that the extent to which the orthography of a given system reflects the phonology of its written words might vary between the different languages, depending on the regularity of the so called “grapheme-to-phoneme consistency” (e.g., Van Orden et al., 1990; Lukatela and Turvey, 1994a,b; Frost, 1998). The degree of such consistency was found to determine the use of the different routes during word recognition, a notion known as the “orthographic depth hypothesis” (Frost, 2005). Indeed, Frost et al. (1987) have observed that the lexical route has little impact in highly transparent orthographies like the Serbo-Croatian one, in contrast to English, where the lexical route is the dominant route for word recognition (Frost, 1998).

The question of the time of occurrence of the various stages of processing (and the routes involved) in visual word recognition in the different orthographies has often been investigated using event related brain potentials (ERPs) during various reading paradigms (see for instance Bentin et al., 1999; Kaan and Swaab, 2003; Simon et al., 2004, 2007; Maurer et al., 2005b, 2008; Grainger et al., 2006; Holcomb and Grainger, 2006; Braun et al., 2009; Briesemeister et al., 2009). One of the tasks used in this context was the lexical decision task (LDT) with pseudohomophone words (hereafter PHw, McCann and Besner, 1987; Seidenberg et al., 1996; Braun et al., 2009). PHw are pseudowords that differ from real words in their orthography but have the same phonology (e.g., *Brane* for the word *Brain* in English). In such tasks, a PHw effect is usually found in terms of longer response time and higher error rate for PHw compared to real words. Using PHw in LDTs, and specifically because of the phonological similarities between the stimuli, enforces the analysis of the orthographic features of the word in order to make the decision (see Ferrand and Grainger, 1992). In ERP studies of visual word recognition, several studies in various alphabetic orthographies have linked different early components with the hypothesized stages of word processing (Bentin et al., 1999; Kaan and Swaab, 2003; Grainger et al., 2006; Holcomb and Grainger, 2006; Simon et al., 2006, 2007; Maurer et al., 2008; Braun et al., 2009; Briesemeister et al., 2009). Most consistently, the N170 component, a negative occipito-temporal response at ~170 ms (Simon et al., 2004, 2006; Maurer et al., 2005a; Bar-Kochva, 2011; Horie et al., 2011; Taha et al., 2013) was linked with the orthographic stage in word recognition. For instance, it was found that stimulus repetition and familiarity modulate the N170 component (Simon et al., 2007). Also, Maurer et al. (2005b) found that the orthographic expertise effects appear around ~170 ms after stimulus onset. In Arabic language, we have recently shown that the words' internal orthographic connectivity modulated both the amplitude and latency of the N170 (Taha et al., 2013). In studies using PHw and real words, ERP differences were also reported during the early components (Newman and Connolly, 2004; Yeung et al., 2004; Grainger et al., 2006; Braun et al., 2009). For example, in a recent study Comesana et al. (2012) analyzed ERPs to examine the role of phonological and orthographic overlap in the recognition of cognate and non-cognate words, conditions that mimicked to some extent the effects of PHw in English and Portuguese. The authors indicated that the differences observed around the P2 component indexed an initial discrimination of the stimuli on the basis of their physical properties. A similar interpretation was proposed by other results with logographic orthographies (Kong et al., 2012). However, in another recent ERP study it was found that responses evoked by PHw differed significantly from those evoked by the real words (taksi vs. taxi) already around 160 ms following stimulus presentation (Braun et al., 2009). This difference which occurred around the time period of the N170 component was explained as expressing an early phonological processing step and not an orthographic one. This interpretation appears to be in contradiction with many other ERP studies which support the notion that this early time window is more related to orthographic processing (see above) and to other studies that suggest that phonological processing occurs later in time. Indeed, it had been proposed that the phonological stage in visual word recognition is reflected in the N320 component, which is measured in mid temporal regions at ~320 ms (Bentin et al., 1999; Simon et al., 2004; Khateb et al., 2007b). This component was found to be modulated by orthographic transparency of the writing system, suggesting that it reflects the sublexical mapping between orthography and phonology (Simon et al., 2006). Regarding the stage of lexical access, which is thought to occur at a later stage after the orthographic and the phonological ones, this has been suggested to involve later components such as the N400 which has repeatedly been linked to lexical-semantic processing (Halgren et al., 2002; Simon et al., 2004; Khateb et al., 2010; Kutas and Federmeier, 2011). Since PHw have the same phonology and semantics as their basic words, it was found that no differences were observed between these conditions around this component (Braun et al., 2009; Briesemeister et al., 2009). Thus, Braun et al. (2009) found that the differences around the N400 were found between words and non-words but not between words and their PHw. In contrast, it has been reported that PHw modulated the P600 component (Vissers et al., 2006), a brain response which had frequently been associated with orthographic error detection and other anomalies. The modulation of the P600 by PHw was interpreted as reflecting a process of monitoring that takes place during language perception and when the cognitive system is found in an indecision state. Support for this notion was recently found in a study on Chinese, a non-alphabetic orthography, where a modulation of the late positive component (600–1000 ms) was reported during orthographic decision and semantic tasks (Kuo et al., 2012). Other researchers suggested that this component is modulated by stimulus familiarity and represents the search for these stimuli in memory, such as with infrequent words (Allan et al., 1998), pseudowords or irregular words (Osterhout and Hagoort, 1998; Shaul, 2011), and as with words that are syntactically inappropriate (Osterhout and Holcomb, 1992; Kaan and Swaab, 2003; Van Herten et al., 2005).

In view of the fact that word recognition in Arabic has scantily been investigated using physiological measures, our objective here was to investigate the time course of word recognition in Arabic with a special emphasis on orthographic processing steps, using an orthographic decision task with real words and PHw. Given the fact that Arabic is an alphabetic language, we expected to replicate previous findings about PHw effects reported in other orthographies. Also, assuming that Arabic has many unique orthographic features, we expected a particular modulation of the early ERP components that are thought to reflect the orthographic stages of word processing. Indeed, the Arabic language has a very particular alphabetic orthographic writing system consisting of 29 letters of which three are long vowels. Short vowels are not considered as part of the alphabet and are represented by diacritical marks added above or below the letters (see Taha, 2013). Most Arabic letters have more than one written form, depending on the letters' position within the written word (in the beginning, middle, or end of word) and on the letters' connectedness with former and subsequent letters (see Taha et al., 2013). In addition, different letters may have the same essential shape and can differ only by the presence (or not) of one or more dots, or by the location of the dots on or below the letter (for example: 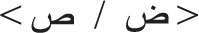 or 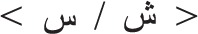 or 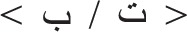. In order to provide the full phonological information in the written Arabic words, these have to be vowelized by diacritical marks (representing short vowels) added above and below the letters within the word. In the case of vowelized written words, the written patterns are considered as shallow orthography, while in the case of non-vowelized written words, the orthography is considered as a deep one. In this later case, which usually appears in texts dedicated to adult readers (Abu-Rabia, 2001), the phonology is not entirely reflected through the orthography and the reader must rely on the context cues to read correctly. Most particularly for our purpose and the task used here, the Arabic phonological system includes a group of phonemes referred to as the “emphatic phonemes.” An emphatic phoneme is one that share a phonological similarity with another phoneme in Arabic and use the same articulation parts of the articulacy system but represented by two different graphemes (for example: the letter 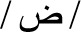 represents an emphatic phoneme, and its similar is the letter 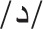 = d, but the 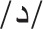 = d itself is not an emphatic one). In Arabic vernaculars (i.e., spoken Arabic dialects), some of these emphatic phonemes are absent within the specific phonological system of certain dialects (for example the emphatic 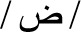 does not exist within some spoken vernaculars). As a main result of the phonological similarity between one emphatic phoneme and its similar (although) non-emphatic phoneme, there are difficulties in spelling words that include one emphatic phoneme or more. Such difficulties appear as inaccuracy in spelling, in the form of phonologically plausible orthographic errors where the subject writes down a PHw instead of writing the correct orthographic pattern of the word (e.g., 
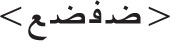
 instead of 
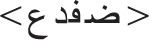
, like the word “kat” instead of “cat” in English). It means that, when making these errors the subject relies on simple phoneme-to-grapheme mapping and not on the specific orthographic knowledge stored in long term memory about such words. Therefore, writing down words that contain those emphatics requires a specific familiarity with the word's orthographic pattern and demands additional cognitive and memory resources. In this regard, it was suggested that difficulty in discrimination between empathic phonemes and their similar non-empathic ones, together with the lack of sufficient orthographic knowledge, is the main reason for producing the abovementioned phonologically plausible orthographic errors during spelling in Arabic (see Abu-Rabia and Taha, 2004, 2006; Taha, 2013). The orthographic decision task used here with real words and PHw, while neutralizing phonological and semantic effects, aimed replicating previous findings about the PHw effects and tracking more specifically visual orthographic processes. Indeed, given the fact that at the phonological level the real words and PHw are identical and at the semantic level they activate the same meanings, we predicted that the discrimination between words and their corresponding PHw would be a relatively difficult task that relies primarily on a careful orthographic analysis. In addition, such a discrimination process, to be efficient, should recruit additional cognitive resources to allow retrieval of orthographic knowledge from long term memory. Therefore, differences in the ERP between real words and PHw were hypothesized to occur during early and late stages of stimulus processing, but not during time periods necessarily devoted to phonological and lexico-semantic processing. At the behavioral level, we expected faster RTs to correctly written words than to PHw.

## Materials and methods

### Participants

Eighteen right handed (15 females and 3 males) Native Arab students were recruited from the University of Haifa to participate in this study using an orthographic decision task during EEG recordings. Their age ranged from 19 into 34 with mean age of 23.4 and *SD* = 3.8. All the participants were right handed with normal reading development and without attention difficulties or other sensory, emotional or neurological disorders. All had normal or corrected-to-normal vision, gave their informed consent prior to the inclusion in the study, and were paid for their participation (35 ILS/h).

### Stimuli and procedure

The stimulus list was composed of 80 real words and their 80 corresponding PHw. The words consisted of 40 concrete literary Arabic nouns and 40 verbs varying from middle to high lexical frequency (mean frequency = 3.65 ± 0.88 on a scale from 1 to 5 by 23 raters). The selected words were between 3 and 6 letters length (mean 4.31 ± 0.79) with an average number of syllable = 2.52 ± 0.82 (range between 2 and 4 syllables). For the purpose of the study, 80 corresponding pseudohomophonic words (PHw) were created. Half of the PHw were produced by replacing a letter in the beginning syllable and the other half by replacing a letter in the last syllable of a real word while keeping the phonology of the word identical [see the following examples: (i) the word 
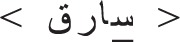
 modified into 
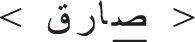
 and (ii) the word 
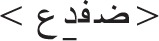
 modified into 
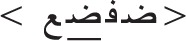
. Taken together, words and PHw totalized 160 stimuli which were pseudorandomly mixed and divided into two experimental blocks each containing 80 stimuli.

Participants were seated comfortably in front of a computer screen, approximately at 90 cm distance and performed a speeded orthographic decision task. Since the main objective of the study was to characterize brain responses involved in the orthographic analysis of the words in Arabic, this task appeared more suitable than a standard LDT in which PHw have to be rejected as non-words while in the mean time they activate the same phonological and semantic processes. Hence, non-words were not used here and the subjects had in the present task only to respond whether or not the presented stimulus was written correctly without implying other phonological and lexico-semantic analysis. Each stimulus was presented for 700 ms on the center of the screen in white over gray background. After each stimulus, they were asked to decide as quickly and accurately as possible using two keyboard keys. The response window was of 1550 ms. The stimuli were written with “Traditional Arabic Fonts” with point size of 45 using the E-Prime v.II software (Psychology Software Tools, Inc., www.pstnet.com/ PA, USA).

### EGG recordings and analysis

Experiments were carried out in an isolated, sound attenuated room. Electroencephalographic (EEG) recordings were collected continuously using a 64 channel BioSemi Active Two system (www.biosemi.com) and the ActiveView recording software (2009). Pin-type electrodes were mounted on a customized Biosemi head-cap, using an electrode gel and arranged according to the 10–20 international system. Two flat electrodes were placed on the sides of the eyes in order to monitor horizontal eye movements. A third flat electrode was placed underneath the left eye in order to monitor vertical eye movements and blinks. The EEG signals were collected reference free (i.e., Biosemi active electrodes), with a 0.25 high pass filter, amplified and digitized with a 24 bit AD converter, at 2048 Hz sampling rate.

ERP epochs were averaged and analyzed offline using the Brain Vision Analyzer software (Brain-products). The EEG data were first filtered (Low pass filter: 30 Hz and High pass filter: 1 Hz), then ocular artifacts were corrected using the Gratton et al. (1983) method and the data were afterwards re-referenced to the common average of all electrodes. The epochs were determined from 100 ms pre-stimulus baseline and 900 ms post-stimulus only for correct responses. Artifacts were rejected (artifacts were defined by amplitudes greater than 50 μV and lower than −50 μV). The resulting data were baseline-corrected for each subject using the 100 ms pre-stimulus interval and then down-sampled to 512 Hz.

### ERP waveshape analysis

In order to characterize response differences between words and PHw, we conducted two analysis. First, we performed a global analysis using point-wise *t*-tests on the individual ERPs of the two conditions using Cartool software© (v.3.43; https://sites.google.com/site/fbmlab/cartool). This aimed at determining time periods and scalp location exhibiting difference between words and PHw. Hence it was performed over all time frames (stimulus onset to 700 ms, i.e., 358 time points) and all recording sites. Time periods that exhibited significant *t*-values (at *p* < 0.05) during at least 5 consecutive time frames (~10 ms) and involved at least three adjacent electrodes, were considered as significant. In the second analysis, and on the basis of previous findings with PHw (see Introduction) and on our results on Arabic orthography (Taha et al., 2013), we compared the amplitude and latency of the N170, P2, and P6 components between conditions. In the analyses presented hereafter, we computed the mean signal for the N170 component in each subject and condition in the time period between 170 and 190 ms from the three left posterior (P7, PO7, and O1) and three right posterior (P8, PO8, and O2) which exhibited the maximum negativity (at PO7) for this component. We then computed the mean amplitude for the P2 component in the time period between 250 and 280 ms from the same electrodes since these were again the ones that exhibited the maximum positivitiy for the component (at PO8, see Figure 2 below). Finally, in view of previous findings regarding the P6 component (see Introduction), the analysis of the late responses were performed around the peak of the P6 component. For this purpose, we computed the mean amplitude from four left central and centro-parietal electrodes (C1, C3, CP3, and CP1) and four right ones (C2, C4, CP4, and CP2) during the time period 450–600 ms. For the analyses of the components' latency, we determined in each subject for the N170 the latency of the most negative time point between 120 and 200 ms and immediately after the most positive time point for the P2 from the same subset of electrodes (as for the amplitude, see above). For the P600, we first computed in each subjects the average of 15 central, centro-parietal and parietal electrodes around Cz, CPz, and Pz which showed the highest P6 amplitude. The resulting individual “P6” waves were then low-pass filtered at 5 Hz [to avoid the selection of spurious peaks, as in Moreno and Kutas (2005), Khateb et al. (2010) for the N400 component] and from these were determined the latency of the most positive peak occurring after 450 ms. Statistical analyses were then conducted on these measures using repeated measures ANOVAs with word condition (word vs. PHw), hemisphere and electrode as within subject factors.

### Source localization analysis

This analysis aimed at estimating the location of the sources in the brain whose activity differentiated the two conditions. Here, we applied LAURA (Grave De Peralta Menedez et al., 2001), a distributed linear inverse solution, to estimate brain regions that lied behind the ERP differences between conditions. This technique, like other distributed inverse solution algorithms, deals with *a priori* unknown number and location of active sources and uses a real head shape model with 4024 solution points in the gray matter. This technique has now been used in a large variety of cognitive paradigms including language tasks (see Ducommun et al., 2002; Ortigue et al., 2004; Blanke et al., 2005; Thierry et al., 2006; Khateb et al., 2007a,b, 2010; Taha et al., 2013). Here LAURA was applied to the topographic maps computed in each subject and condition, from the mean signal of the periods of interest. The individual inverse solutions were first averaged to display the mean source localization over subjects and then were compared statistically using paired *t*-tests with the significance level fixed at *p* < 0.01. Sources localization were then reported using the Talairach and Talairach's 1988 *x, y, z* coordinates.

### Behavioral analysis

The mean of the individual reaction times and the individual rate of correct responses were computed separately of the words and the PHw conditions. These values were compared statistically using paired *t*-tests.

## Results

### Behavioral measures

The individual means of the RTs and the rate of correct responses were computed for each subject in each condition. Responses with RTs below 250 ms were excluded from the individual mean responses. The paired *t*-test comparing the individual performance in words and PHw showed no significant difference (*p* = 0.55, mean = 86 ± 10.8% and 83 ± 19.4% respectively). The comparison of the RTs revealed significantly faster responses for words than for PHw (*t* = 0–3.0, *df* = 17, *p* < 0.009, mean = 729 ± 124 and 784 ± 147 ms respectively). The comparison of the accuracy and the RTs for the PHw where the letters in the real word were changed in the first and in the last syllables (while keeping the phonology of the word) showed no significant difference both in terms of accuracy and RTs. Similarly, no difference between these two types of PHw was also observed when comparing the individual standard deviations of the RTs.

### Electrophysiological analysis

**Figure 1:**
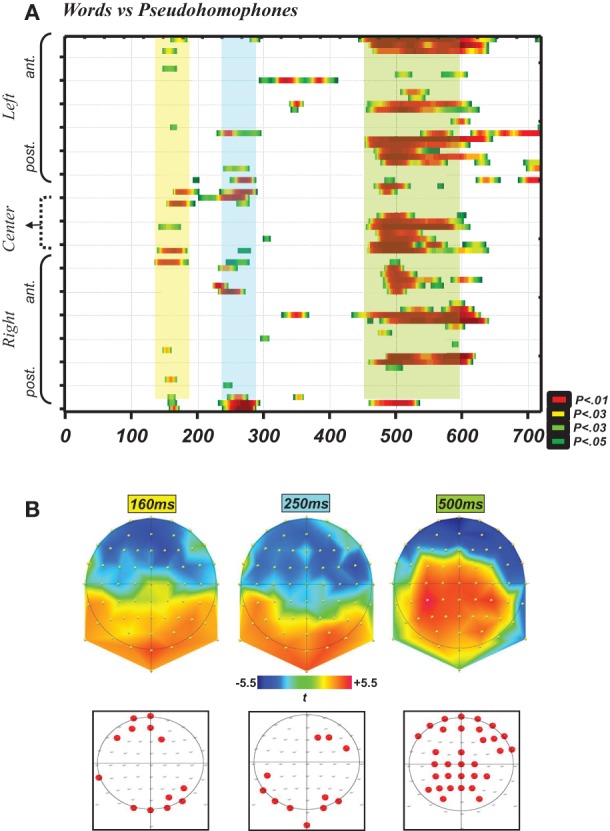
**(A)** Graph depicting the statistical *p*-values (yellow-green for *p* < 0.05; dark red for *p* < 0.01 and highest values) of the exploratory point-wise *t*-tests analysis (see Methods) performed on all time points from 0 to 700 ms (i.e., 358 time frames, *x*-axis) and all 64 recording sites (*y*-axis) for the comparisons words vs. PHw. This analysis showed that robust (highest *p*-values) and consistent (longest in duration and implying many electrodes) differences appeared already in the N170 time window, then at ~250 ms (i.e., P2 component) and then around the P600 component. **(B)** This panel illustrates *t*-maps (upper raw) successively for the time periods at 160, 250, and 500 ms (see the same color scale for *t*-values). The lower raw presents on schematic maps the location of the electrodes depicting significant differences during these time points, and shows that during the N170 there were six adjacent posterior and six anterior sites with significant *p*-values (lower left) and in the other periods there was also a high number of significant and adjacent electrodes.

Due to technical problems during EEG recordings and to the presence of a high amount of artifacts in other subjects, three subjects were excluded and the analysis presented here concerns 15 subjects. As indicated in the Methods section, the first analysis using point-wise *t*-tests on all time frames and electrodes aimed at identifying time periods and locations where the electrophysiological signal differed between words and PHw. This analysis is presented in Figure 1, which depicts graphically in A the significant *p*-values (at *p* < 0.05) on all electrodes and over all time frames (for 10 ms consecutively) up to 700 ms post-stimulus. It shows that the earliest differences appeared around the time window of the N170 component. The significant differences appeared then at around 250 ms and then between around 500 ms, with the latter differences extending also after 750 ms. The upper row in Figure 1B displays the *t*-maps successively for the first period (N170), then immediately after for the second period (referred hereafter to as the P2, at 250 ms) and finally for the late period (hereafter the P6, between 450 and 600 ms). The lower raw in Figure 1B shows the location of the electrodes with significant differences. These schematic maps indicate that: (i) the differences around the N170 concerned mainly posterior sites (with six adjacent electrodes, appearing a little more in the right) and frontal sites (again six adjacent electrodes, appearing a little more on the left), (ii) the P2 differences concerned again bilateral sites (although more dominantly in the left) and (iii) the P6 differences involved a high number of electrodes distributed mainly centro-parietally and bi-frontally. The later differences, appearing at around 750 ms onwards and being of lesser interest for our purpose, were not further analyzed here.

**Figure 2:**
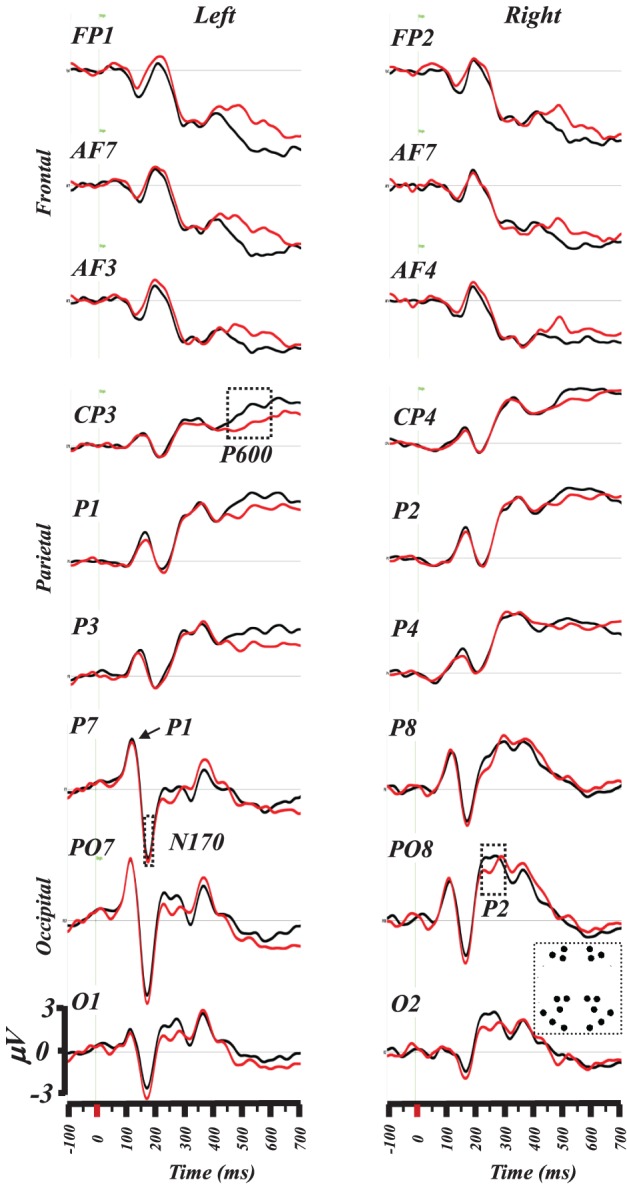
**Superimposition of grand-mean ERP (from −100 to 700 ms post-stimulus) induced by words (black traces) and PHw (red traces).** The selected electrodes represent antero-posteriorly distributed left and right electrodes where differences were maximally evident for the N170 and P2 components at electrodes PO7 and O1 (left) and PO8 and O2 (right). Note the succession of the P1and the N170 components on P7. The differences during the P6 component are best illustrated on centro-parietal electrodes (see CP3). The inset in lower left depicts the location of the selected electrodes (see text for details).

Figure 2 illustrates a superposition of the grand-mean ERP traces (from −100 to 700 ms) on a subset of frontal, parietal and postero-occipital electrodes that exhibited maximal response differences between words and PHw. This illustration indicates that the difference in amplitude for the N170 and the P2 involved particularly the left PO7 and O1 sites and the right PO8 and PO2 ones. For the P6, the differences appeared on various centro-parietal sites including here at CP3, P1, CP4, and P2. In the following analysis, we further quantified the difference between words and PHw by computing the mean amplitude (and the latency) for each of these components.

### The N170 component

**Figure 3:**
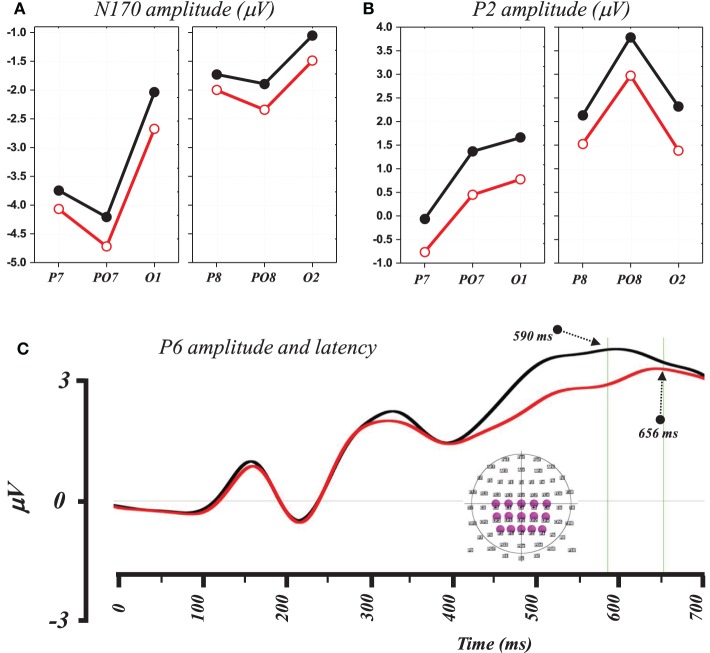
**(A)** Graph showing the mean N170 amplitude at electrodes P7, PO7, and O1 (left) and P8, PO8, and O2 (right) where hemisphere (left vs. right) and condition effects were statistically observed (see text). **(B)** Graph showing the mean P2 amplitude at the same left and right electrodes where hemisphere (left vs. right) and condition effects were statistically observed (see text, black lines for words and red for PHw). **(C)** Traces of the averaged ERP signal from 15 centro-parietal electrodes to illustrate differences in amplitude and peak latency of the P6 in words (black) and PHw (red). The inset in lower right depicts the location of the selected electrodes for this illustration.

The 2 × 2 × 3 ANOVA performed on the N170 amplitude (computed between 170 and 190 ms) using word condition (Word vs. PHw), hemisphere (left vs. right) and electrode (3 sites: P7, PO7, and O1 in the left and their parallel P8, PO8, and O2 in the right) as within subject factors showed significant main effects of condition [*F*_(1, 14)_ = 5.39, *p* < 0.04], hemisphere [*F*_(1, 14)_ = 7.61, *p* < 0.02], and electrode [*F*_(2, 28)_ = 7.45, *p* < 0.003]. As illustrated in Figure 3A, the condition effect was due to higher N170 amplitude in PHw than in words. The hemisphere effect was due to a greater N170 negativity in left hemisphere electrodes. The electrode effect was due to varying N170 amplitude across the different electrodes with PO7 showing the largest negativity in the left and PO8 in the right. The comparison of the N170 peak latency computed from these two electrodes, with the largest negativity, was performed with a 2 × 2 ANOVA using condition and hemisphere as within subject factors. No significant main effect of word condition was observed (mean = 160 ± 9 and161 ± 10 ms respectively for words and PHw) and no interaction with hemisphere, which showed a significant effect [*F*_(1, 14)_ = 5.36, *p* < 0.04], due to earlier latency in the right.

### The P2 component

A 2 × 2 × 3 ANOVA was performed on the P2 amplitude computed (between 250 and 280 ms) from the same electrodes as the N170 using again word condition (Word vs. PHw), hemisphere (left vs. right) and electrode (3 sites) as within subject factors. This showed a significant main effects of condition [*F*_(1, 14)_ = 16.36, *p* < 0.002], of hemisphere [*F*_(1, 14)_ = 8.44, *p* < 0.02], and electrode [*F*_(2, 28)_ = 5.43, *p* < 0.02]. No interaction was observed between condition and the other factors but a significant interaction was found between hemisphere and electrode [*F*_(2, 28)_ = 6.87, *p* < 0.004]. As shown in Figure 3B, the condition effect was due to a larger P2 amplitude in words (mean = 1.97 mV) than in PHw (mean = 1.06 mV). The hemisphere effect was due to a greater positivity in right than in left hemisphere electrodes. The electrode effect was due to varying P2 amplitude across the different electrodes with PO8 showing the highest amplitude in the right. Here also, the peak latency of the P2 component was computed from PO8 and PO7. The 2 × 2 ANOVA performed on the P2 peak latency values using condition and hemisphere as factors showed no significant main effect of word condition (mean = 227 ± 8 and 225 ± 7 ms respectively for words and PHw) and no interaction was found between this factor and the hemisphere.

### The P6 component

A 2 × 2 × 4 ANOVA was performed on the P6 amplitude, computed on the mean signal between 450 and 600 ms, using word condition (Word vs. PHw), hemisphere (left vs. right) and electrode (4 sites: C1 C3 CP3 CP1 from the left and C2 C4 CP4 CP2 from the right) as within subject factors. This showed significant main effects of condition [*F*_(1, 14)_ = 21.53, *p* < 0.0005] due to higher P6 positivity in words (mean = 2.88 mV) than in PHw (mean = 2.04 mV). In addition, there was an electrode effect but no interaction was observed between condition and the other factors. The electrode effect was mainly due to the fact that more centro-posterior electrodes showed globally higher amplitude than central ones. The comparison of the latency of the P6 computed (as the most positive time point after 450 ms) from the average of 15 centro-parietal electrodes around Cz showed that this component peaked significantly earlier in words than in PHw (see Figure 3C, *t* = −2.73, *df* = 14, *p* < 0.02, mean = 590 ± 70 and 657 ± 63 ms respectively).

### Source localization analysis

**Figure 4:**
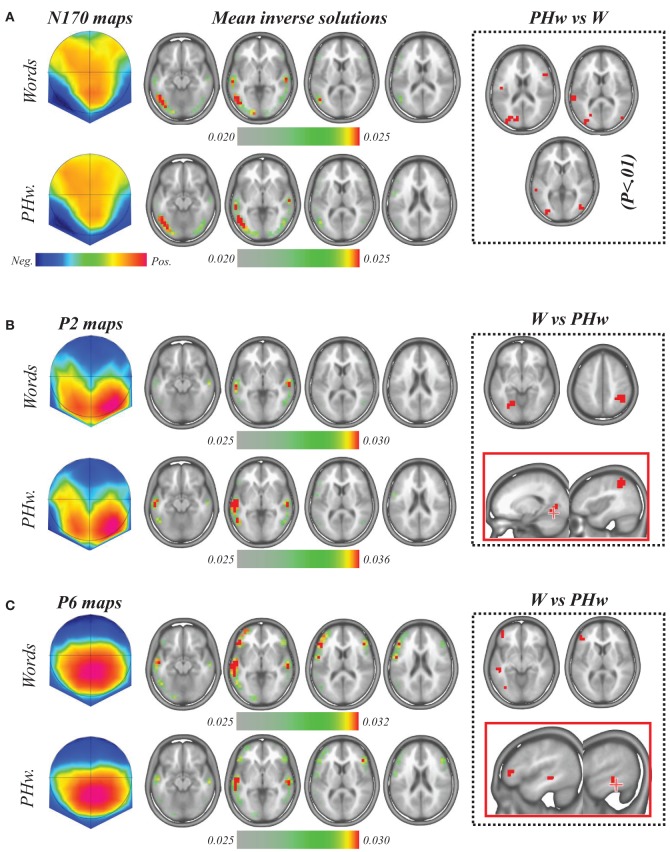
**(A)** Topographic potential map (left) and average source localization computed during the N170 period from the individual inverse solution in words (upper raw) and PHw (lower raw). In both cases, maximal activity involved left temporo-occipital areas. Paired *t*-tests comparing inverse solutions to PHw vs. words (significance at *p* < 0.01, right dashed square) showed more activity for PHw in the left posterior areas (see text). **(B)** Topographic potential map (left) and average source localization computed during the P2 period in words (upper raw) and PHw (lower raw). In both cases, maximal activity was found in bilateral temporal areas. Paired *t*-tests comparing words vs. PHw showed more activity for words (right dashed square) in the left lingual gyrus. **(C)** The same analysis performed during the P6 period on the individual inverse solutions showed a dominant pattern of source in the left hemisphere. Paired *t*-tests comparing words vs. PHw showed more activity for words (right dashed square) in left frontal, temporal and occipital areas. The sources estimated by LAURA inverse solution are displayed on four successive MRI slices where the maximal activity was observed. Note that the inverse solutions in **(A,B)** are scaled to their maximum (see color scale for each raw). In the right panels (dashed squares) the red color corresponds to areas where solution points (i.e., voxels) showed significant statistical differences at *p* < 0.01.

In order to estimate which brain areas where at the origin of the ERP differences that differentiated words and PHw, we applied the LAURA linear inverse solution (Grave De Peralta Menedez et al., 2001) to the time periods of interest in each subject and condition and compared these solutions statistically (see Figures 4A–C). First, for the N170, the individual inverse solutions were computed from each subject mean signal between 170 and 190 ms (as for the amplitude analysis) and these were then compared using paired *t*-tests to determine the differences between PHw and words. As shown in Figure 4A which depicts the mean inverse solution for the N170, source maps indicated that this period maximally involved in both words and PHw the left temporo-occipital areas in particular an extensive recruitment of the left inferior temporal gyrus and middle occipital gyrus (BA 19/37, Talairach x, y, z coordinates = −47, −58, 0 and −41, −70, −4) and slightly the left lingual gyrus (BA 17). Also, bilateral sources were found in the superior and middle temporal gyrus (BA 21/22, −59, −33, 3 and −59, −23. −2). The statistical comparison between PHw vs. words (since the N170 was larger in PHw, Figure 4A, right panel) showed more activity for PHw in the left post-central gyrus (BA 43, −47, −10, 18), left superior temporal gyrus (BA 22, −59, −34, 14), left Cuneus (BA 18, −11, −80, 22) and the bilateral middle occipital gyrus (BA 19, −29, −80, 22), but also in right middle frontal gyrus (BA 46, 46, 18, 23).

For the P2 and the P6 components, the individual inverse solutions were computed from each subject mean signal between 250 and 280 ms and between 460 and 600 ms respectively. As illustrated in Figure 4B, the mean inverse solution for the P2 showed that both in words and PHw, the maximal activity was found in the superior temporal gyrus bilaterally (BA 22, Talairach *x, y, z* coordinates = −58, −23, 3), together with another weaker activity extending posteriorily into the bilateral middle temporal gyrus (BA 37/19, −53, −58, 0). The statistical comparison between words vs. PHw (since the P2 was larger in words) showed significantly more activity for words (B, right panel, *p* < 0.01) in the right inferior parietal lobule (BA 40, 41, −44, 48) and in the left lingual gyrus (BA 19, −17, −64, −5). For the P6 period (Figure 4C), a bilateral pattern of activity with a left dominance was also observed, including the left superior frontal gyrus (BA 10, −35, 58, −1), the inferior frontal gyrus bilaterally (BA 47, −47, 29, 0), the superior temporal gyrus bilaterally (BA 22, −59, −17, 2), the bilateral inferior temporal gyrus (BA 19, −47, −58, 0) and the left lingual gyrus (BA 17, −17, −87, 1). The statistically significant differences between words vs. PHw (since the P6 was larger in words) showed more activity for words only in the left hemisphere (see Figure 4C, right panel), including the inferior frontal gyrus (BA 46, −41, 35, 5), middle frontal gyrus (BA 11, −41, 46, −10), middle temporal gyrus (BA 20, −53, −35, −6) and inferior occipital gyrus (BA 19, −35, −69, 0).

## Discussion

In this study, we sought to investigate the time course of word recognition in Arabic using ERP analysis with a special emphasis on orthographic analysis processes. For this purpose, we used an orthographic decision task with real words and PHw. We predicted to find word type effects in terms of RT and to observe ERP differences mainly during the early and late stages of processing. Our results replicated previous findings and showed a clear effect of PHw in RTs which were significantly faster to words than to PHw. Also, our results replicate previous observations regarding the modulation of the late responses (P6 component) and extend them to emphasize the modulation of the early responses (N1-P2 components) involved in orthographic processing steps. The use of the PHw paradigm was motivated by the fact that their phonological similarity with the real words forces the reader to analyze the orthographic features of each stimulus in order to make the correct decision. Accordingly, we also postulated that, together with late serial analytic processes, early automatic orthographic processes might be involved in this judgment task. Since PHw differ from real words in their orthography but have the same phonology and lead to the same meaning, the discrimination between words and their corresponding PHw is supposed to rely on the analysis of the physical properties of the stimulus (i.e., the orthography). Here, PHw differed from the real words just in one graphemic feature but shared the same phonology and the remaining majority of their graphemes. This fact is supposed to enable testing if the discrimination process begins early (i.e., during the early visual recognition steps), or if there are phonological processes involved in later stages. Due to the fact that words and PHw had the same phonology and activate the same semantic meaning, differences in the ERP were not expected during periods devoted to phonological or lexical-semantic processing.

The ERPs analysis supported our major assumptions and revealed that the discrimination between real words in Arabic and their PHw occurred already in the early stages where the readers assess the orthographic features of the presented words. This was reflected by a significant modulation of N170 component whose amplitude increased in the PHw condition. The N170 component was hypothesized to represent the processing step in word reading and recognition related to the orthographic stage [see Taha et al. (2013) for a recent discussion]. The differences observed here cannot be explained in terms of low level physical characteristics of the stimuli such as stimulus length or the spatial frequency (Bar-Kochva, 2011; Horie et al., 2011), since these were similar in length and only one grapheme was changed. Other studies have reported a modulation of the N170 by word frequency (being larger for frequent than infrequent words and pseudowords), and interpreted this result as evidence that the N170, although reflecting probably a prelexical orthographic processing, could for frequent words represent a more holistic process allowing words to be processed as a global visual pattern (Simon et al., 2007). Here, one could postulate that the orthographic patterns of the real words represent the situation of the frequent orthographic patterns while the PHw represent the irrgular and non-frequent patterns. In this case, the finding that PHw induced larger N170 is hardly understandable under the previous explaination of word frequency where one would have expected it to be larger for real words. One plausible interpretation here is that the larger N170 in PHw was due to the fact that these necessitated a deeper visual orthographic analysis together with increased cognitive/attentional ressources for determining the locus of the orthographic error. In terms of the timing of these early differences, the result observed here is in line with other ERP investigations which showed that differences between real words and PHw (Braun et al., 2009) and between words and scrambled words (Zhang et al., 1997) occurred as early as 150 ms after stimulus presentation. Although in the recent study reported by Braun et al. (2009) the early ERP differences between PHw and real words were attributed to phonological activation, we assume that, since both types of stimuli had similar phonological patterns, this earlier modulation could only be attributed to difference in orthographic analysis. This postulation is in accordance with other studies that reported differences in the latency and distribution of the N170 during visual orthographic tasks where this component, as observed here, was found larger over the left than the right hemisphere sites (Sereno et al., 1998; Bentin et al., 1999; Hauk and Pulvermuller, 2004; Maurer et al., 2005b; Appelbaum et al., 2009; Grossi et al., 2010). The source localization analysis, which aimed at shedding light on the cerebral origin of the scalp recorded responses, showed that the estimated brain activation maps explained quite nicely the first steps of visual word processing as suggested by some computational models and previous functional imaging studies. For instance, the local combination detectors (LCDs) model proposed by Dehaene et al. (2005) suggests that the visual recognition of words depends firstly on the detection of the local orienting bars which are the basic components of the letters. After responding to specific letters, the system responds to multi letter strings (i.e., morphemes and small words), a process which seems to rely on the activation of the left occipital-temporal system, thought also to be involved in object recognition (Dehaene et al., 2005). The source localization analysis performed here during the N170 period are compatible with this model's assumption and indicated, as illustrated here the involvement mainly of the occipito-temporal areas during the processing of both words and PHw (see Jobard et al., 2003; Taha et al., 2013).

The early process of analysis of the physical orthographic features during visual word recognition went beyond the N170 component since amplitude differences were measured also at the level of the P2 component, where words induced higher responses than PHw. This results fits with previous observations comparing word and scrambled-words and revealing long-lasting ERP differences which started after 170 ms post-stimulus and remained up to 600 ms (Zhang et al., 1997). This finding related to the P2 is also compatible with others from a recent study (Comesana et al., 2012) using electrophysiological and behavioral measures, which examined the role of phonological and orthographic overlap in the recognition of cognate and non-cognate words. The authors suggested that the differences observed around the P2 component indexed an initial discrimination of the stimuli based on their physical properties. The modulation of the ERPs on posterior sites during the P2-N2 components (around 250 ms post-stimulus) had also been reported in other studies with different types of stimuli including faces and objects (see Pegna et al., 2008). This modulation was attributed to processes of conscious visual recognition (Koivisto et al., 2006) or visual perceptual closure (Doniger et al., 2000) and represent the earliest manifestation of awareness. In a previous ERP mapping study (Khateb et al., 2002), it was found that the map segments (i.e., ERP microstates) occurring immediately after the N170 component differentiated words and pseudowords by exhibiting shorter duration in words than in non-words (but also in images than in scrambled images). This time period was interpreted as corresponding to a phase of enhanced pattern analysis where stimuli are re-evaluated before the recognition process and final decision are operated in successive distinct steps. The differences observed here around the N1-P2 complex (N170 and P200) on the posterior sites support the notion that the differentiation of the written Arabic words starts early and depends on intensive visual discrimination process (Taha et al., 2013). The reliance during this period clearly on visual discrimination process might be explained by the fact that the particular characteristics of the Arabic orthography (i.e., mainly the fact that different forms might represent almost each letter and that certain letters differ only by the presence or absence of a dot or more) oblige the readers to develop automatic and sophisticated visual discrimination mechanisms. The fact here that the accuracy level in PHw did not differ significantly from that for words seems to support this notion of enhanced visual verification processes in Arabic. The source localization maps estimated during the P2 component in both words and PHw involved bilaterally the superior and middle temporal gyrus, with significant differences appearing maximally and most interestingly in the left lingual gyrus. This later area has already been involved in visual word recognition (Mechelli et al., 2000a,b) and its activation was shown to increase with word length. Also, it has been reported that activity in the lingual gyrus decreases with increasing stimulus duration (Price and Friston, 1997). Taken together, these observations indicate that this region participates to the visual analysis of the stimulus and probably to word recognition as a familiar orthographic pattern. The difference in terms of activated areas during the P2 suggested also the involvement of the inferior parietal lobule (BA 40). This part of the parietal cortex has been involved in vigilant attention and recently proposed to be part neither of the conventionally “dorsal” nor conventionally “ventral” system, but a non-spatial parieto-frontal circuit that plays a role in top-down processing (Husain and Nachev, 2007). Thus, the involvement of this part of the attentional system might be explained by the specific features of the Arabic orthographic system which necessitates increased cognitive demands due to the physical similarities between the letters and their changing forms according the place in the word (see Taha et al., 2013).

Because of the similar phonology between words and PHw, no differnces were expected and thus were found during the processing stages asociated with the phonological discrimination steps previously described around the N320 component (Bentin et al., 1999). This component was generally measured during visual word recognition in mid temporal regions at ~320 ms (Bentin et al., 1999; Simon et al., 2004; Khateb et al., 2007b). For instance, in a previous ERP investigation by Khateb et al. (2007b) using a phonological judgment task, response differences between rhyming and non-rhyming words occurred already around 300 ms post stimulus with increased negativity for rhyming words over left temporal sites. Also, differences in the analysis of the semantic and phonological content of the words were reported in a previous study during the time window between 280 and 380 ms after stimulus presentation. Our global analysis showed no difference during these periods (i.e., the 300 ms time range) devoted to phonological processing and that the differences observed were probably not related to phonology. Similarly, as predicted, no difference was found during the time period of the N400 component, classically devoted to the semantic processing of the words and sentences (Kutas and Federmeier, 2011).

The higher amplitude and the earlier latency of the P6 (P600) component might interpreted as an index of late memory monitoring processes before and around the decision making (Kaan and Swaab, 2003). The difference in the peak latency (about 67 ms) of this component, which probably explains the amplitude differences at centro-parietal electrodes, closely resembles the difference in the mean RT between words and for PHw (~55 ms). It has previously been suggested that the late components associated with anomalies and error detection like the P6 component reflect deep processing rather than automatic discriminations processes (Kaan and Swaab, 2003) and are typically found at centro-parietal electrodes (Osterhout and Holcomb, 1992). Here, the modulation of the P6 could be interpreted as reflecting a monitoring stage during orthographic analysis that enables correct decisions about the errors and anomalies detected in the orthographic patterns of written words in long term memory (Allan et al., 1998; Osterhout and Hagoort, 1998; Van Herten et al., 2005; Vissers et al., 2006; Shaul, 2011). During this period, the estimated source maps pointed to a left dominant activation that comprised the classical language areas such the inferior frontal gyrus and the superior temporal gyrus, all involved in reading (Fiez and Petersen, 1998). The significant differences between words and PHw were found only in the left hemisphere including in the inferior frontal gyrus. A greater activation in language areas including the inferior frontal gyrus is often observed in functional studies when testing both for word frequency and lexicality effects (Price, 2010; Woollams et al., 2010). The difference of activation between words and PHw in such language areas during this specific time period might be explained by the earlier response latency in words relative to PHw, which attests of a more efficient and rapid way to identify and read the correct stored orthographic patterns. Actually, one can assume that such areas, as indicated by the source estimation maps, are involved in the reading of both correctly written and erroneously written words, but the time course of this activation is reached earlier in words, hence the difference in the response amplitude.

To summarize, the present research highlight the importance of a dominant visual discrimination stage during the word recognition in Arabic, especially when these words are to distinguish from homophones that share the same phonology and lead automatically to the same meaning. This finding supports our previous findings that stressed the important role of early automatic visual discrimination during word recognition in Arabic (Taha et al., 2013). The differences observed here at the level of the N1-P2 complex and then later during the P6 (together with the lack of differences during time periods related to phonological and lexico-semantic processing) suggests that words recognition in Arabic, possibly more than in other orthographies, begun with serial orthographic-phonemic assembly processes. The late response differences, which replicate those found in previous studies, are thought to reflect error monitoring and memory processes before decision making. The findings presented here are the first of their kind which assess the time course of visual word recognition in the Arabic language using an orthographic decision task. Further research using different paradigms are still needed to better explore the brain mechanisms involved in the visual recognition of word in this particular orthography. Thus, it would be of interest to verify how the differences observed between words and PHw compare with effects lexical frequency among readers of Arabic.

## Author contributions

The first author Haitham Taha designed the experiment, collected the EEG data, performed ERP averaging and preliminary analysis, and wrote the first draft of the article. The second author Asaid Khateb completed the analysis of the ERP data, performed source localization and improved the draft, especially the results section and the discussion.

### Conflict of interest statement

The authors declare that the research was conducted in the absence of any commercial or financial relationships that could be construed as a potential conflict of interest.
